# Shotgun proteomic analysis of quail liver under feed deprivation and low-energy diet conditions

**DOI:** 10.1016/j.vas.2026.100813

**Published:** 2026-08-11

**Authors:** Doha Mohamad Khalifeh, Gabriella Gulyás, Renáta Knop, Gebrehaweria K. Reda, Gergő Kalló, Csaba Szabó, Éva Csösz, Levente Czeglédi

**Affiliations:** a Department of Animal Science, Institute of Animal Science, Biotechnology and Nature Conservation, Faculty of Agricultural and Food Sciences and Environmental Management, 138 Böszörményi Street, University of Debrecen, Debrecen, 4032 Hungary; b Doctoral School of Animal Science, University of Debrecen, 138 Böszörményi Street, 4032, Debrecen, Hungary; c Department of Evolutionary Zoology and Human Biology, University of Debrecen, Debrecen, 4032, Hungary; d Department of Animal Science, College of Agriculture and Environmental Science, Adigrat University, Adigrat, Ethiopia; e Proteomics Core Facility, Department of Biochemistry and Molecular Biology, Faculty of Medicine, University of Debrecen, 4032 Debrecen, Hungary; f Department of Animal Nutrition and Physiology, Institute of Animal Science, Biotechnology and Nature Conservation, Faculty of Agriculture and Food Sciences and Environmental Management, University of Debrecen, 4032 Debrecen, Hungary

**Keywords:** Poultry, Proteomics, Feed intake, Metabolism, Liver

## Abstract

Feed intake determines nutrient utilization and production efficiency in poultry, yet hepatic mechanisms underlying responses to acute nutritional challenges remain insufficiently characterized. This study aimed to identify the liver proteins and pathways associated with feed intake regulation in Japanese quails (*Coturnix japonica*) subjected to different nutritional challenges. Eighteen 12-wk-old male quails (245.20 g ± 0.213) were housed individually and allocated to ad libitum control, 24 h feed-deprived, or 24 h low metabolizable energy groups (n = 6/group). The basal diet contained 18% crude protein and 12.13 MJ/kg energy, whereas the low-energy diet had 6.30 MJ/kg and 11.60% crude protein. Liver proteins were extracted, identified, quantified, combined with STRING-based functional analysis. In total, 856 proteins were identified, of which 563 met the filtering criteria. Compared with the control, the low-energy diet induced 85 differentially abundant proteins (DAPs) through increasing oxidative phosphorylation, fatty acid β-oxidation, mitochondrial substrate supply, and RNA-binding/translation, with decreased ER-Golgi trafficking, intracellular transport, and glycolysis-associated proteins. Feed deprivation yielded 81 DAPs by emphasizing mitochondrial respiratory chain assembly and oxidative phosphorylation, lipid oxidation, and detoxification, while suppressing ribosomal biogenesis, protein turnover, and fed-state metabolic processing. Direct comparison had 76 DAPs and revealed that low energy maintains active cytoplasmic translation/ribosome biogenesis and xenobiotic metabolism, while feed deprivation enriches carboxylic acid metabolism and mitochondrial protection against oxidative stress. Quail liver demonstrated distinct proteomic responses to energy reduction compared with complete feed withdrawal. Notably, oxidative phosphorylation and lipid utilization emerged as central adaptive mechanisms and potential biomarker pathways in response to nutritional stress.

## Highlights

• Quail liver shows unique proteomic response to energy reduction vs starvation.
• Low-energy diet upregulated oxidative phosphorylation, but reduced glycolysis.
• Feed deprivation enriched mitochondrial activity, and suppress ribosome biogenesis.
• Oxidative phosphorylation and lipid utilization are potential biomarker pathways.

## 1 Introduction

Feed intake is one of the most fundamental and significant metrics for evaluating the quality of a reproductive animal population, and improving its efficiency is critical (Gao et al., 2025; Wen et al., 2021). External environmental conditions, internal physiological traits of the individual animal, and feed composition parameters primarily impact feed intake, which can significantly affect the overall performance (Nyachoti et al., 2004).

While transcriptomics and genomics provide information on gene expression and genetic potential, proteomics bridges the gap to phenotype by revealing post-translational modifications and actual protein activity. This is crucial because protein abundance and function often diverge from mRNA levels, especially in complex traits such as feed intake and metabolism (Burgess, 2004; Richards, 2003). Proteomic studies have indicated that dietary interventions (e.g., probiotics, mineral supplementation) alter hepatic and intestinal protein profiles, improving nutrient metabolism and feed efficiency (Jarosz et al., 2022; Zampiga et al., 2018; Zheng et al., 2016). Studies have examined the proteomic profiles of poultry skeletal muscle and duodenum to understand feed metabolism (Kaewsatuan et al., 2022; Kong et al., 2016; Zampiga et al., 2018). Most research articles have tested feed intake regulation in poultry as a response to different nutritional statuses in the brain (hypothalamus) (Jozsa et al., 2006; Saneyasu et al., 2022), intestine (jejunum) (Simon et al., 2019), or other digestive organs (proventriculus, gizzard) (NAKASHIMA et al., 2013; Simon et al., 2017). However, there is limited understanding of how this regulation occurs in the liver, which is a known metabolic organ.

In poultry, as in mammals, the liver plays an important role in a diverse array of metabolic and homeostatic functions, serving as a biochemical center responsible for the majority of synthesis, homeostasis, and detoxification processes (Zaefarian et al., 2019). It plays an important role as an exocrine gland in digestion via bile secretion, carbohydrate and protein metabolism, and lipid synthesis. The liver also plays an endocrine role by synthesizing various proteins, including those present in the bloodstream, enzymes, hormones, and factors involved in coagulation and immune response (Denbow, 2015). Maintaining optimal liver conditions is imperative for ensuring avian health. A comprehensive understanding of the metabolic status of the liver during the feed intake response is crucial for enhancing poultry production strategies and maximizing animal performance by targeting potential biomarkers. Japanese quail (*Coturnix japonica*) is used as an avian model for investigating metabolic mechanisms relevant to poultry species because of its rapid growth, short reproductive cycle, and ease of handling. In addition, the major of physiological functions and metabolic pathways of the liver are highly conserved between chicken and quails, making findings from chicken studies a valuable framework for understanding hepatic responses to nutritional challenges in quail (De Lima et al., 2025; Ishige et al., 2016; Pu et al., 2020; Shu et al., 2025). We hypothesized that acute feed restriction and exposure to a low-energy diet would induce distinct hepatic proteomic adaptations in Japanese quail by remodeling pathways involved in energy production, mitochondrial function, and lipid metabolism to maintain energy homeostasis under nutritional stress. We aimed to investigate the differentially abundant liver proteins and core regulatory pathways in quails subjected to different nutritional challenges using proteomic analysis.

## 2 Materials and methods

### 2.1 Animals and experimental design

The study was conducted according to the guidelines of the European Union Directive 2010/63/EU on the protection of animals used for scientific purposes. The experiment was conducted in the animal house of the Institute of Animal Science, Biotechnology, and Nature Conservation, University of Debrecen.

We selected 18 male quails at 12 weeks of age with similar body mass (245.20 g ± 0.213) and randomly allocated them to individual cages (30 cm × 22 cm × 40 cm, length × width × height) using the randomized block design for one week to acclimate them to the experimental conditions, with each group consisting of six birds (n = 6). The cages were housed within the same controlled environment, maintaining a temperature of 23–25 °C, relative humidity (RH) of 60–65%, and a 12L:12D During the acclimation period, the animals were allowed free access to the breeder feed and water. The study consisted of three experimental groups: an ad libitum control group, a feed-deprived group for 24 h, and ad libitum low metabolizable energy diet group for 24 h. The average feed intake after 24 h experimental period was 22.15 g for control group, and 12.73 g for the low energy group, whereas birds in the feed-deprived group had no access to feed during the treatment period.

### 2.2 Feed formulation

The basal (control) breeder feed was prepared according to the National Research Council (Nutrient Requirements of Poultry: Ninth Revised Edition, 1994) recommendations for quails on a corn-wheat-soybean as the primary ingredients, containing 18% crude protein and 12.13 MJ/kg metabolizable energy. The low metabolizable energy diet consisted of 50% sunflower hull and 50% designed less metabolizable energy diet. To prepare the low-energy diet, sunflower hulls were finely ground to obtain a texture close to the basal feed. The ground hulls were then thoroughly mixed with the basal diet to ensure homogeneity before administration to the birds. The details of the feed composition are presented in Table 1.

**Table 1:** Ingredient composition and nutrient level of the breeder basal and low metabolizable energy diet.

Ingredients, %	Basal feed	Low metabolic Energy feed
Sunflower hull	0	50
Corn	25	7.48
Wheat	10.1	12.25
Wheat bran	10	5
Pea	13.02	12.5
Soybean meal	24.86	8.69
Oil	9.07	0
Limestone	5.83	2.94
MCP	0.96	0.45
L-Lys	0	0.03
L-Met	0.19	0.1
L-Thr	0.07	0.04
Leucine	0.01	0.04
Isoleucine	0.005	0.02
Valine	0.005	0.02
Salt	0.38	0.19
Vitamin and mineral premix^a^	0.5	0.25
*Total*	*100.00*	*100.00*
**Nutrient content, %**		
ME, MJ/kg	12.13	6.30
Crude protein	18 (18.2*)	11.60
Crude fiber	3.98	28.39
Lys	1.014	0.67
Met	0.45	0.30
Thr	0.74	0.49
Trp	0.216	0.16
Leu	1.42	0.84
Ile	0.77	0.48
Arg	1.26	0.75
Leu/Ile	1.84	1.75
Ca	2.5	1.44
P	0.608	0.35
Na	0.15	0.08

a 1 kg premix provided: 1000,000 IU/kg vitamin A, 200,000 IU/kg vitamin D_3_, 4900 IU/kg vitamin E, 200 mg vitamin K_3_, 150 mg vitamin B_1_, 500 mg vitamin B_2_, 1200 mg Ca-d-Pantothetane, 400 mg vitamin B_6_, 2 mg vitamin B_12_, 11 mg biotin, 2502 mg niacin, 60 mg folic acid, 300,000 mg choline chloride, 13,200 mg Zn, 1920 mg Cu, 9612 mg Fe, 13,200 mg Mn, 180 mg I, 42 mg Se, 12 mg Co. MCP: monocalcium phosphate. *tested in the department laboratory.

### 2.3 Tissue sampling and protein extraction

After 24 h, the birds were euthanized by cervical dislocation, and the liver tissue was removed, placed immediately into liquid nitrogen, and stored at −80 °C until subsequent analysis.

The liver tissue was homogenized with a mortar and pestle using liquid nitrogen. The powdered tissue (50 mg) was transferred to a sterile tube, and 500 µL of lysis buffer (8.5 M urea, 2 M thiourea, 4% (w/v) CHAPS, 30 mM Tris) was added. The mixture was then incubated for 60 min on ice, vortexed every 10 min, and centrifuged at 10,000 g for 20 min; the supernatant was collected and stored at −80 °C until further analysis. Protein concentration was determined using Bradford reagent (SERVA Electrophoresis GmbH, Heidelberg, Germany).

### 2.4 Proteomic sample preparation

Proteins (50 µg) were denatured with 6 M urea for 30 min; thereafter, reduced with 10 mM dithiothreitol (Bio-Rad, Hercules, CA, USA) at 56 °C for 60 min and further alkylated with 20 mM iodoacetamide (Bio-Rad, Hercules, CA, USA) in the dark for 45 min. Before trypsin digestion, the samples were diluted with 25 mM ammonium bicarbonate (Sigma-Aldrich, St. Louis, MO, USA) to decrease the urea concentration to 1 M. Trypsin digestion was performed at 37 °C overnight by adding MS-grade modified trypsin (ABSciex, Framingham, MA, USA) at a 1:25 enzyme-to-protein ratio. The digested proteins were dried in a speed-vac and dissolved in 1% formic acid. The samples were desalted with C18 PierceTips (Thermo Scientific, Waltham, MA, USA), dried, and re-dissolved in 1% formic acid before mass spectrometry analyses, and 5 µg of the digested sample was spiked with iRT peptide mixture according to the manufacturer’s instructions (Biognosys, Zurich, Switzerland).

### 2.5 Mass spectrometry analysis

Prior to mass spectrometric analysis, the peptides were separated in a 50-min water/acetonitrile gradient using an Easy nLC 1200 nano UPLC (Thermo Scientific, Waltham, MA, USA). The peptide mixtures were desalted in an ACQUITY UPLC Symmetry C18 trap column (20 mm × 180 µm, 5 μm particle size, 100 Å pore size, Waters, Milford, MA, USA), followed by separation on a bioZen Peptide XB-C18 analytical column (75 µm x 150 mm, 2.6 µm particle size; Phenomenex, Torrance, CA, USA). Solvent A was 0.1% formic acid in LC-MS grade water (Sigma, St. Louis, MO, USA) while solvent B was 95% acetonitrile (Sigma, St. Louis, MO, USA) containing 0.1% formic acid (Sigma St. Louis, MO, USA). The flow rate was set to 700 nL/min. Mass spectrometry analyses were performed using a Nanospray Flex ion source in positive ion mode. The spray voltage was set to 2300 V, while the sweep gas was 0.2 L/min and the transfer capillary temperature was 300 °C. MS1 scans were recorded by the Orbitrap analyzer in a 350–1650 *m/z* scan range. The resolution was set to 120,000, and the AGC target value was 1 × 106. MS1 spectra were recorded in profile mode. After MS1 scans, 27% HCD collision energy was used for ion fragmentation after precursor ion isolation in the quadrupole. For MS2 scans, the scan range was between 200–2000 m/z, and the MS2 spectra were recorded in centroid mode in the Orbitrap (the resolution was set to 30,000 and the AGC target value was set to 1 × 106). The data was collected using the data-independent acquisition (DIA) method, with 33 scan windows. In each case six biological replicates and two technical replicates were examined.

### 2.6 Data analysis

For DIA data analysis, we used DIA-NN (v1.8.1) (Demichev et al., 2019); the DIA raw data were aligned against the *Coturnix japonica* subset of the UniProt database (downloaded December 2024). The following settings were applied: FASTA digest for library-free search, missed cleavages set to 2, maximum number of variable modifications set to 2, methionine oxidation and N-terminal acetylation added, and carbamidomethylation of cysteine added as a fixed modification. Proteins were accepted if they were identified with at least two peptides. Protein quantification was performed based on the MaxLFQ values, and the data were normalized to iRT peptides. The DIA-NN outputs were filtered for duplicates. In some cases, multiple accession numbers http://www.ncbi.nlm.nih.gov/entrez/query.fcgi?cmd=searchdb=pdbdoptcmdl=genbankterm=wwwere assigned to a protein; in these cases the 1st accession was kept. We applied another filtration: we kept those proteins for further statistical analysis that were present in at least 6 technical samples out of 12 per group.

### 2.7 Identification of differentially abundant proteins (DAPs)

DIA quantification intensity values were transformed to a logarithmic scale with a base of 2. All statistical analyses were performed using R version 4.5.0 (http://www.r-project.org/).

Venn diagrams were generated using the *eulerr* package to visualize the common and unique proteins. Heatmaps were visualized using the *pheatmap* function provided by the ‘*pheatmap’* package (version 1.0.13). To obtain heatmaps for proteins uniquely present in one group (only NAs in the other group), NA values were imputed as zeros.

To assess the DAPs, we considered (i) those proteins that were present in at least 6 samples from a group and were missing in all samples of the other group and (ii) those that showed statistically significant changes between the groups.

We used the Limma package in R to determine differentially abundant proteins (DAPs) (p adj. <0.05, using the Benjamini-Hochberg (BH) test) in the protein expression between the control, low-energy, and feed-deprivation groups.

### 2.8 DAPs network analysis

To examine the protein-protein interaction networks, the STRING platform (version 12.0, http://string-db.org) using the Japanese quail (*Coturnix japonica*) database was used, considering a high confidence score of 0.7 for interaction (Minina et al., 2022; Szklarczyk et al., 2023; Yu et al., 2023) with no >50 interactors at the 1st shell (Rengaraj et al., 2018). Biological process (Gene ontology), molecular function (Gene ontology), cellular component (Gene ontology), Kyoto Encyclopedia of Genes and Genomes (KEGG), and Reactome pathway enrichment analyses were performed using STRING. Pathways with a similarity of ≥ 0.8 were merged, with a minimum count in the network of 10. GO-enriched proteins, KEGG, and Reactome pathways were considered enriched with an FDR ≤0.05.

## 3 Results

### 3.1 Liver proteome identification

A total of 856 proteins were initially identified by high-throughput proteomics analysis after eliminating unnecessary or replicated protein identifications. Summary information about the mass spectrometry analysis is provided in Supplementary Table 1 and Supplementary Figures 1–3. Of the 856 identified proteins, 563 were present in at least 6 samples in each group out of 12 samples.

### 3.2 Control vs. low-energy

**Fig. 1:**
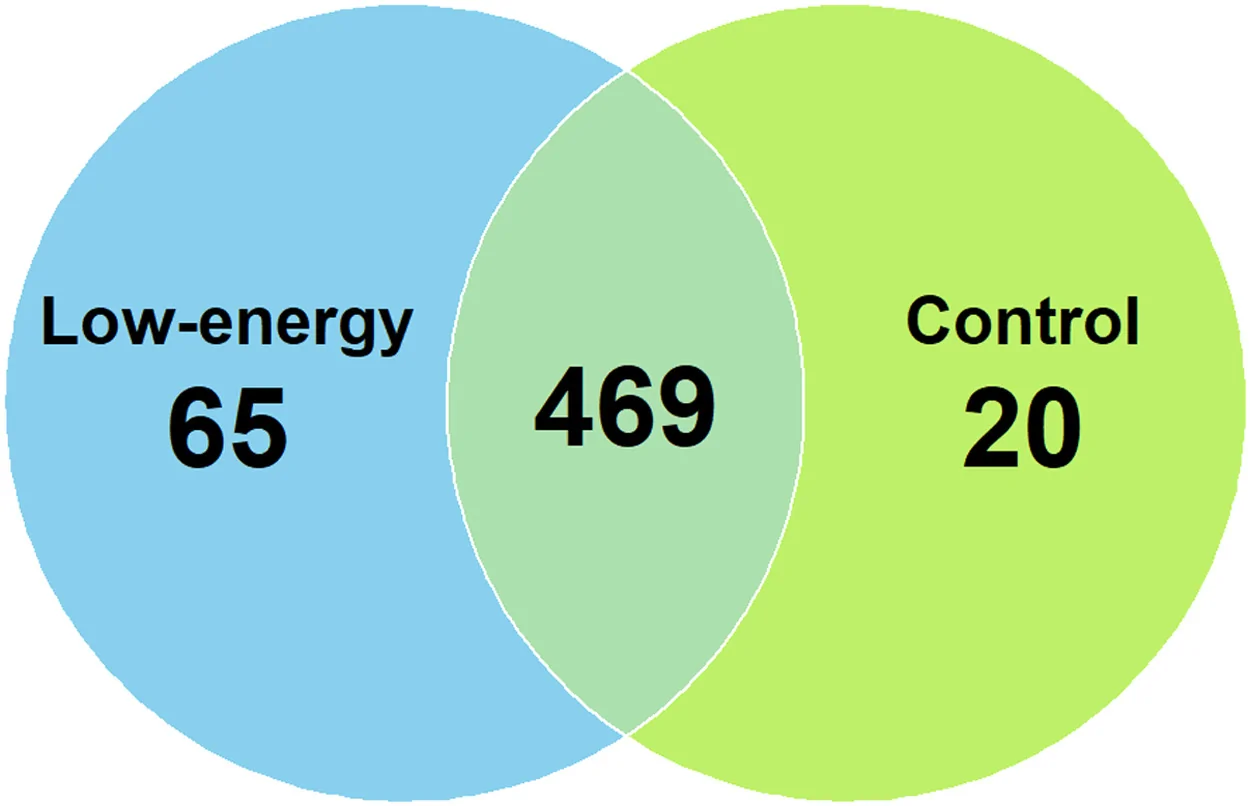
A Venn diagram illustrating the identified liver proteins that were unique to the low-energy group (left) and the control group (right), or common to both groups (center).

**Fig. 2:**
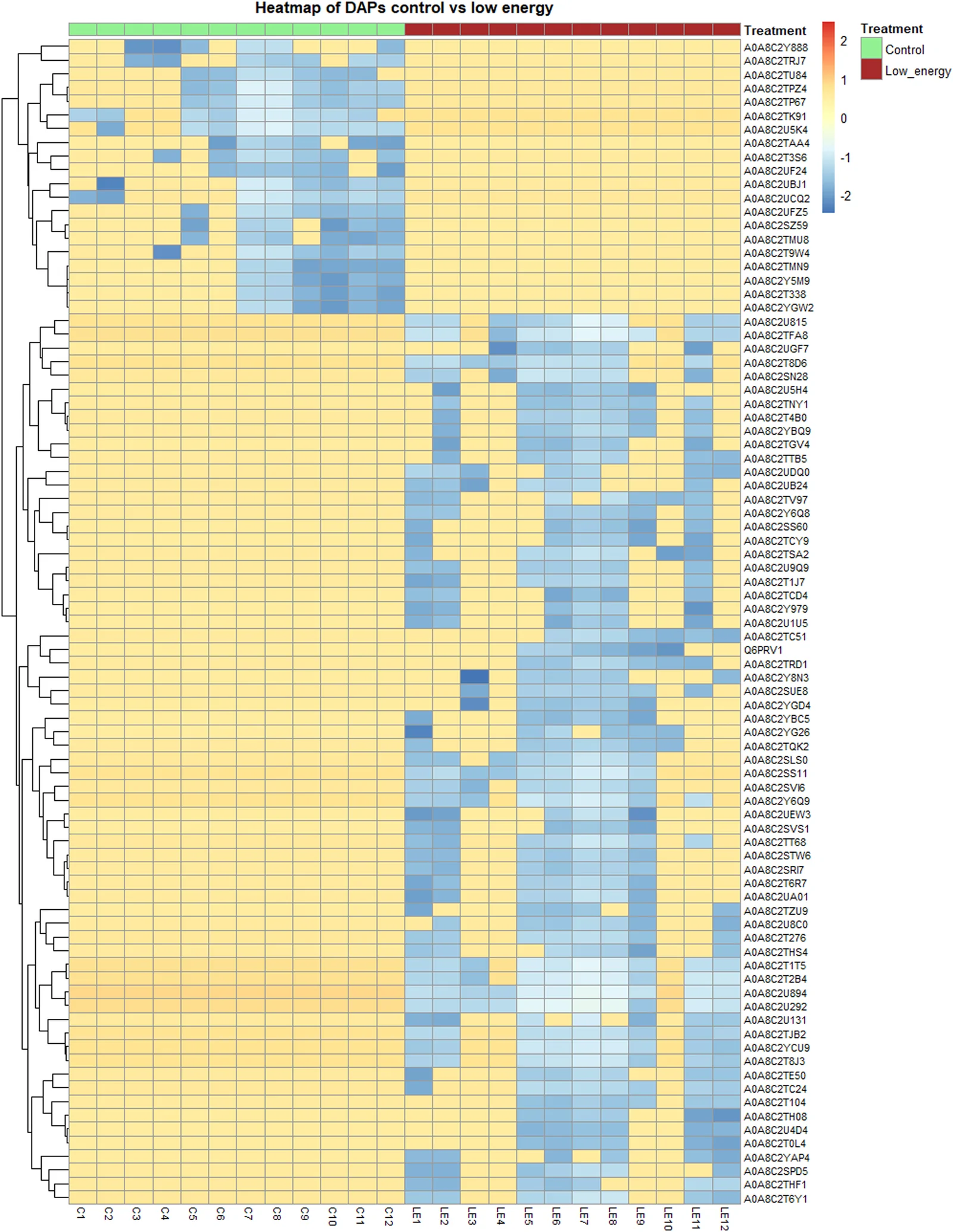
Heatmap of differentially abundant proteins (DAPs) in the control and low-energy groups. The expression levels were visualized using a gradient color. Heatmap was generated using duplicate technical runs from 6 biological replicates per group (n = 12 technical runs/group).

Of the 563 filtered proteins, 554 were present in the control and low-energy groups (Fig. 1). We identified 85 DAPs, 65 of which were more abundant in the low-energy group and 20 of which were less abundant (Fig. 2, Supplementary Table 2).

**Fig. 3:**
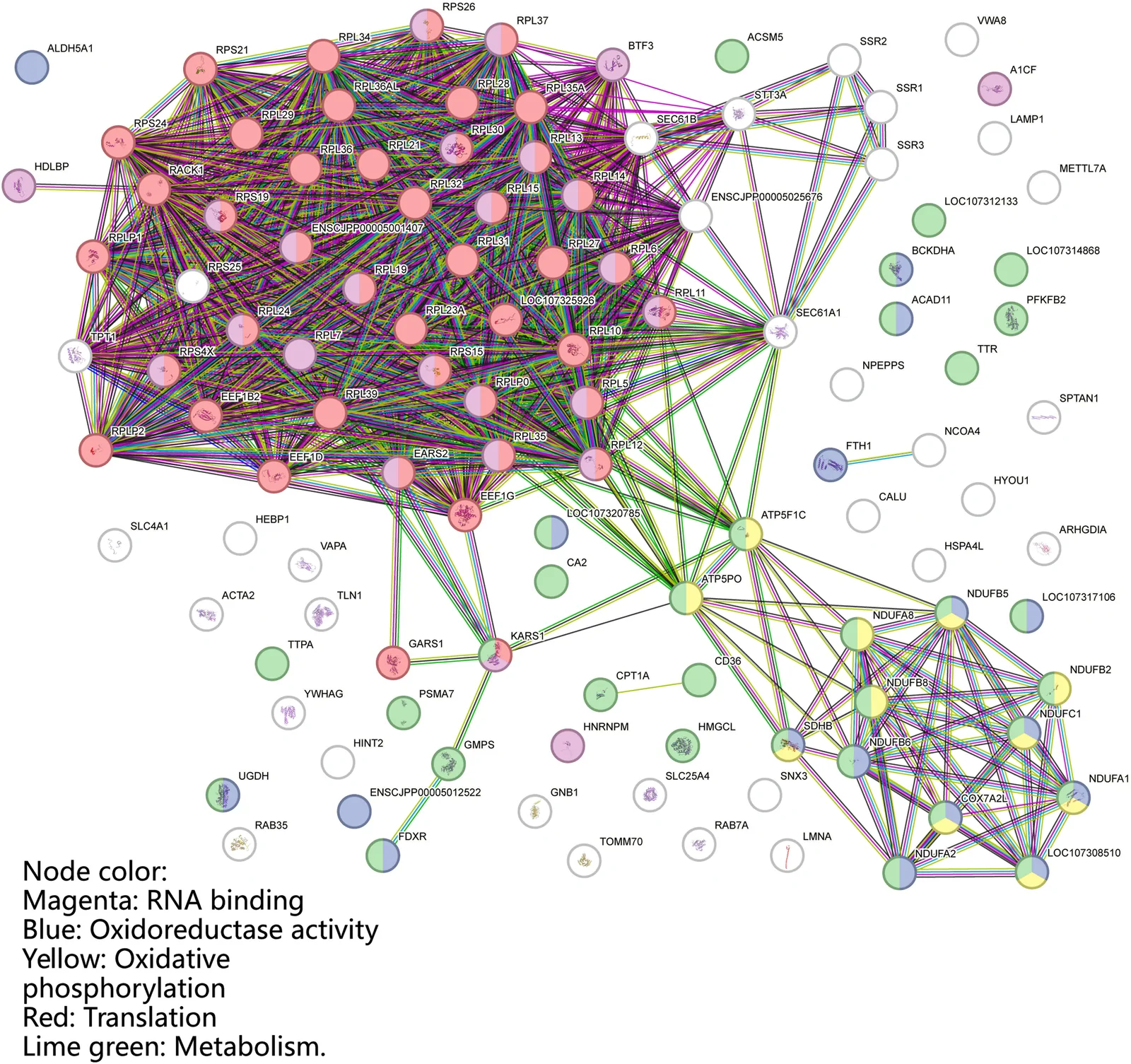
Protein-protein interaction network of differentially abundant proteins with higher abundance in the low-energy group (control vs. low-energy comparison), using STRING. Node colors are based on the functions.

The functional enrichment analysis showed that the DAPs with high abundance in the low-energy group were related to RNA binding (GO:0003723), oxidoreductase activity (GO:0016491), translation (GO:0006412), oxidative phosphorylation (cjo00190), and metabolism (MAP-1430728), indicating a coordinated hepatic metabolic adaptation to altered energy availability (Fig. 3).

**Fig. 4:**
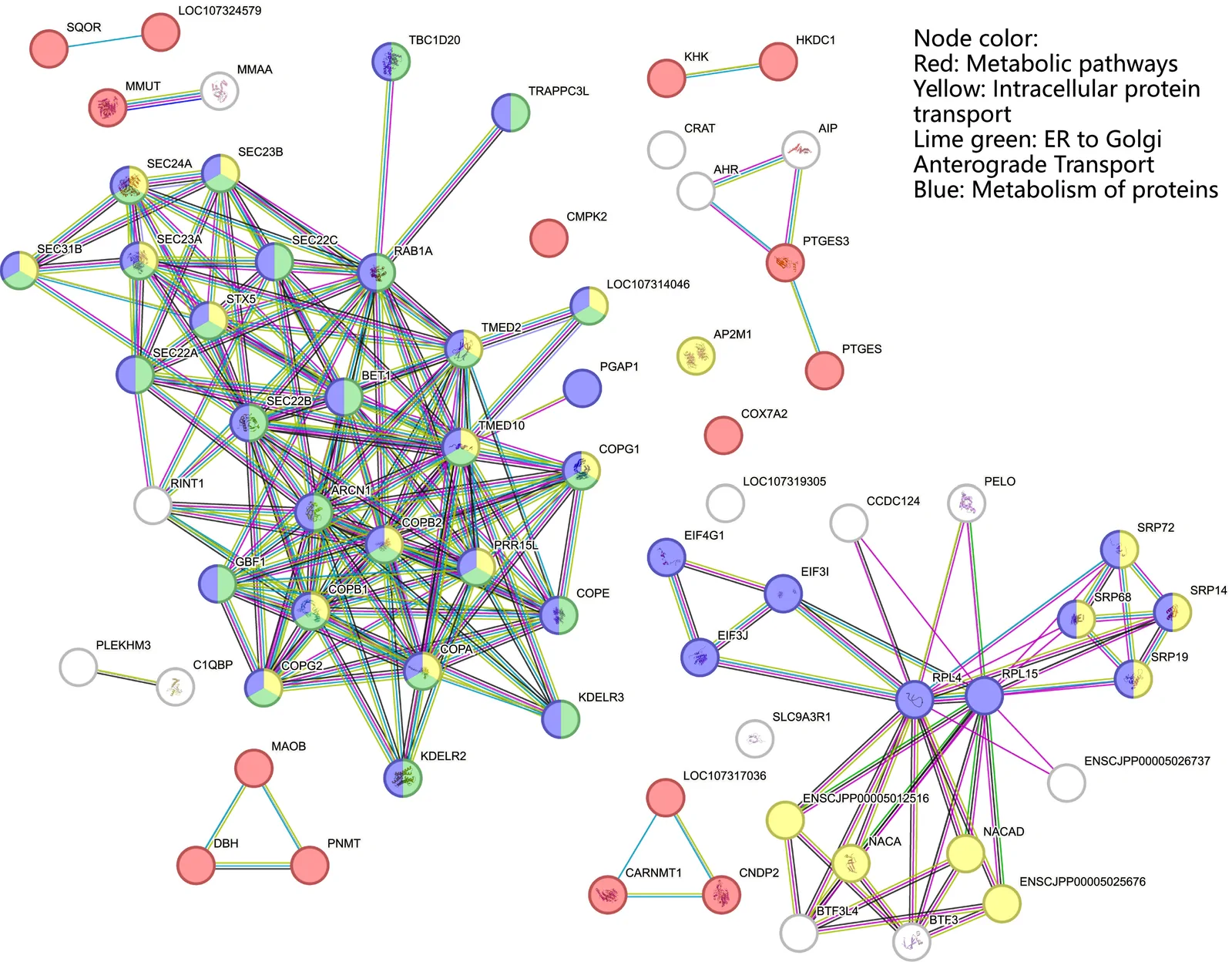
Protein_Protein interaction differentially abundant proteins with lower abundance in the low-energy group (control vs. low-energy comparison), using STRING. Node colors are based on the functions.

In the set of 20 DAPs with lower abundance in the low-energy group, the enriched pathways were related to intracellular protein transport (GO:0006886), metabolism of proteins (MAP-392499), metabolic pathways (cjo01100), and ER to Golgi anterograde transport (MAP-199977) (Fig. 4).

### 3.3 Control vs. feed-deprived group

**Fig. 5:**
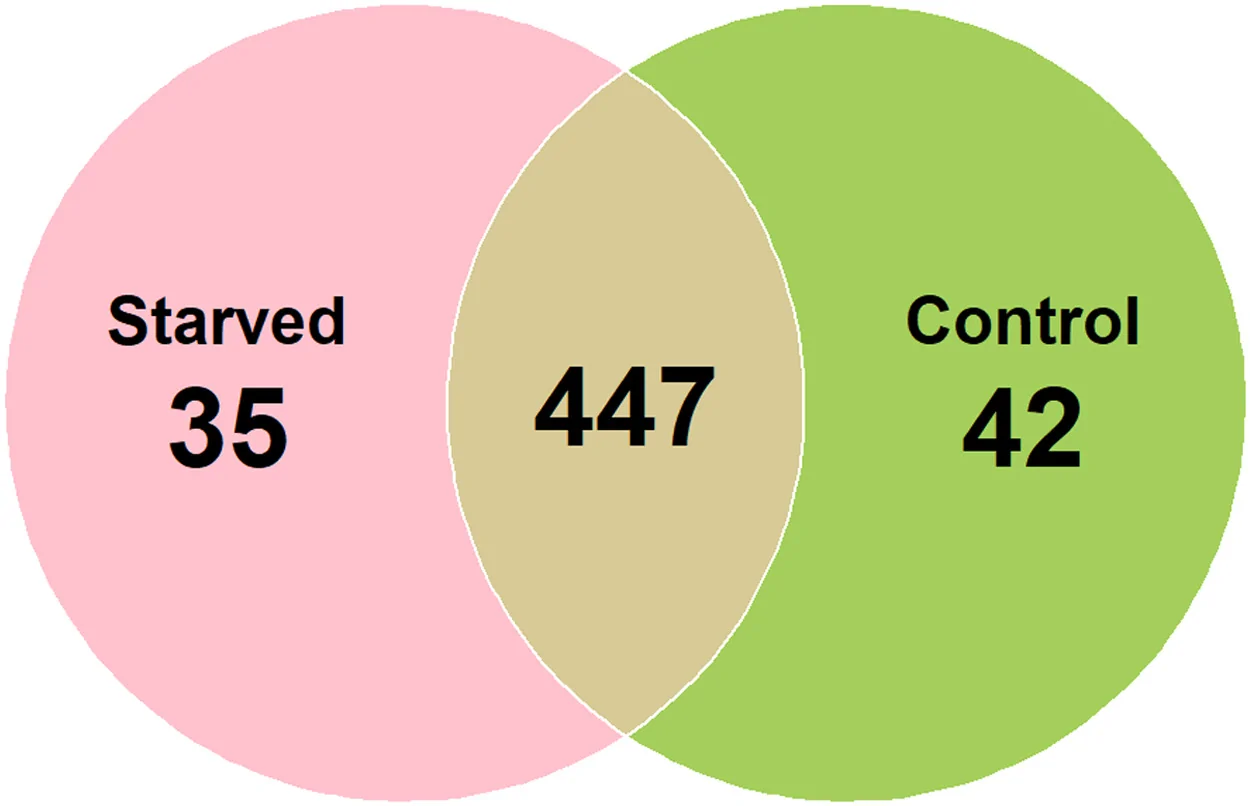
A Venn diagram of the proteins identified in the liver that are unique to the feed-deprived group (left) and the control (right), or were common to both groups (center). Starved: feed-deprived group.

**Fig. 6:**
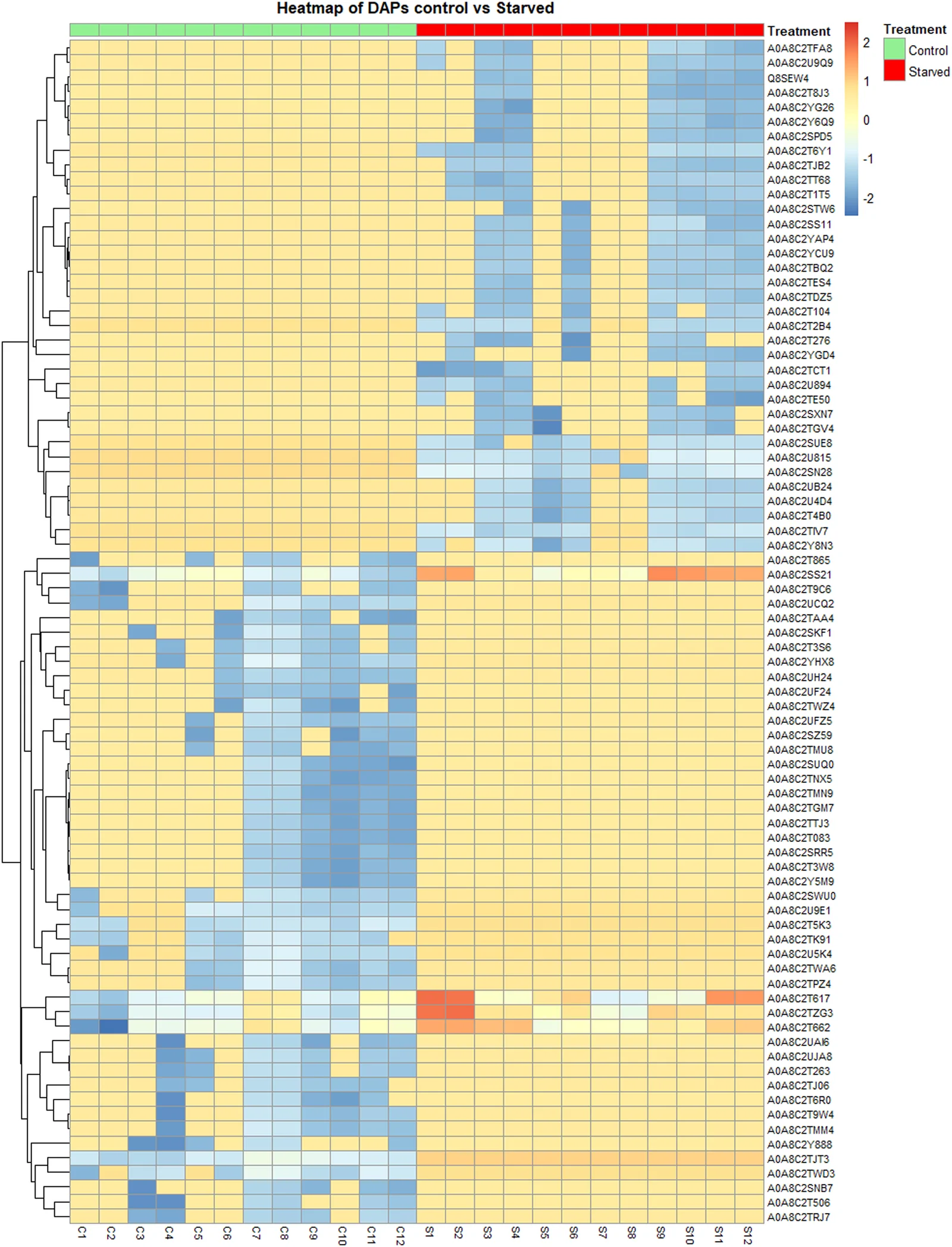
Heatmap of differentially abundant proteins (DAPs) in the control and feed-deprived groups. The expression levels were visualized using a gradient color. Starved: feed-deprived group.

The total number of proteins in the filtered list for the feed-deprived and control groups was 524 (Fig. 5). Of the 81 DAPs between the control and feed-deprived groups, 35 were uniquely expressed in the feed-deprived group, 42 in the control group, and 4 showed statistically significant differences in protein abundance (p.adj. <0.05) (Fig. 6, Supplementary Table 2).

**Fig. 7:**
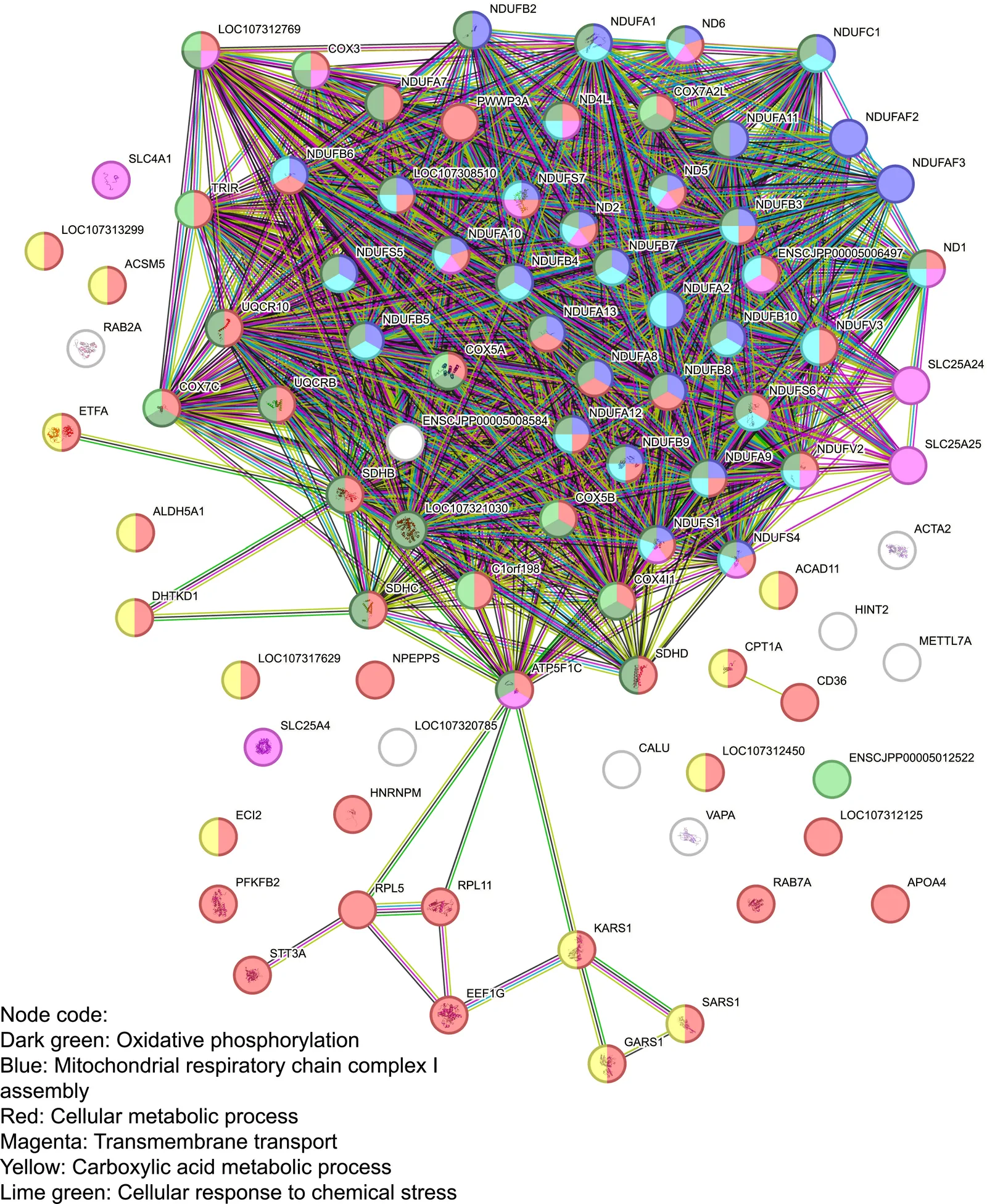
Protein_Protein interaction of differentially abundant proteins with higher abundance in feed-deprived group (control vs feed deprived comparison), using STRING. Node colors are based on the functions.

Functional analysis showed that in the DAPs with higher abundance in the feed-deprived group, the protein set was enriched for pathways related to mitochondrial respiratory chain complex I assembly (GO:0032981), cellular metabolic process (GO:0044237), transmembrane transport (GO:0055085), carboxylic acid metabolic process (GO:0019752), oxidative phosphorylation (cjo00190), and cellular response to chemical stress (MAP-9711123), indicating a hepatic metabolic shift to producing energy from different sources due to feed deprivation (Fig. 7).

**Fig. 8:**
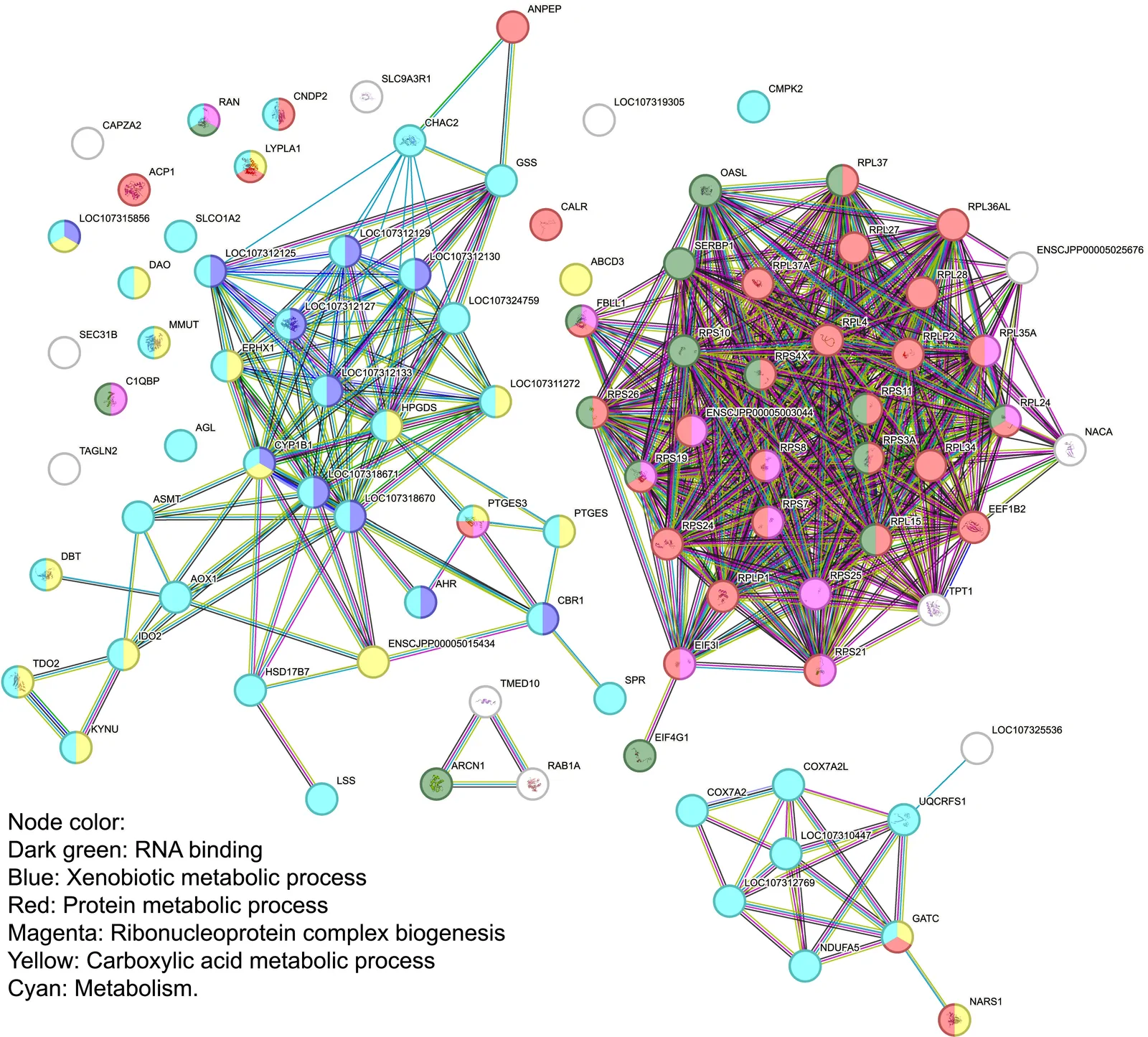
Protein_Protein interaction of differentially abundant proteins with lower abundance in the feed-deprived group (control vs feed deprived comparison), using STRING. Node colors are based on the functions.

In the set of 42 proteins with lower abundance in the feed-deprived group, the enriched pathways were related to protein metabolic process (GO:0019538), RNA binding (GO:0003723), xenobiotic metabolic process (GO:0006805), carboxylic acid metabolic process (GO:0019752), and metabolism (MAP-1430728) (Fig. 8).

### 3.4 Low-energy vs. feed-deprived

**Fig. 9:**
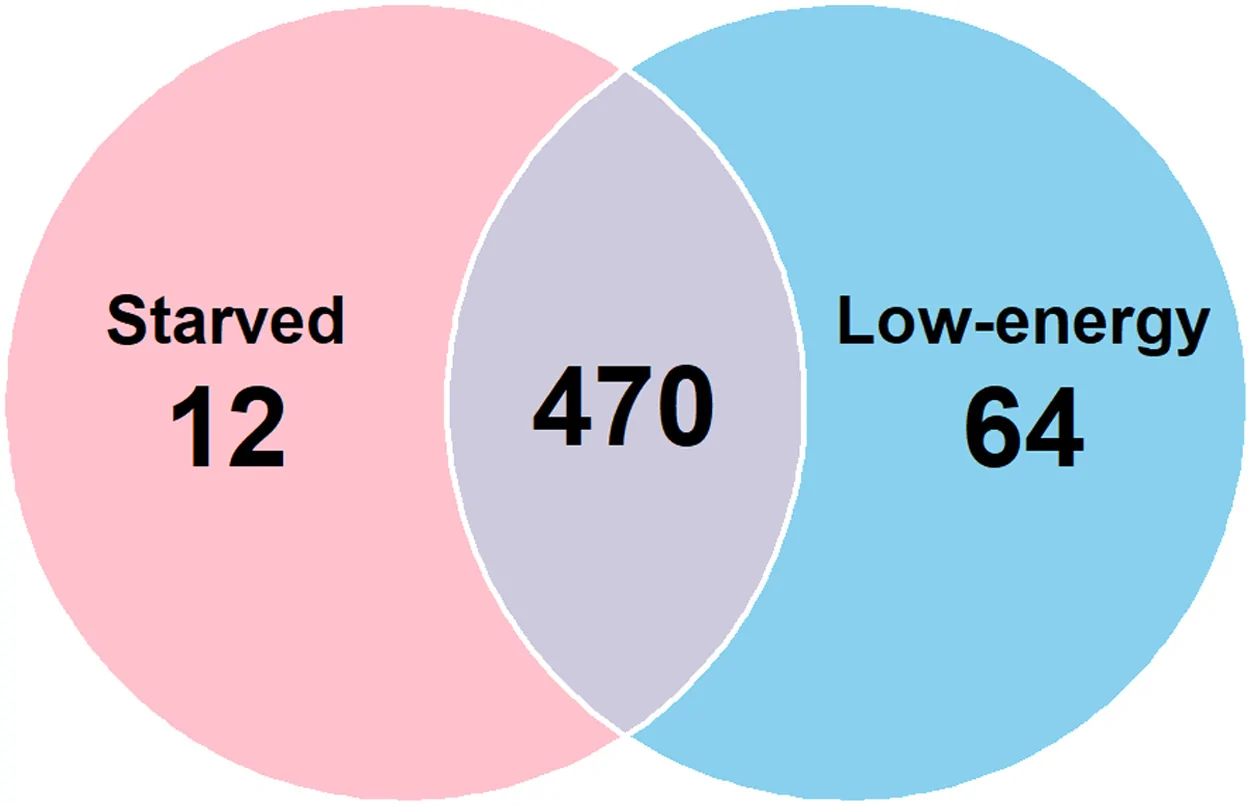
A Venn diagram showing the identified liver proteins that were unique to the feed-deprived (left) and the low-energy group (right) or common to both groups (center). Starved: feed-deprived group.

**Fig. 10:**
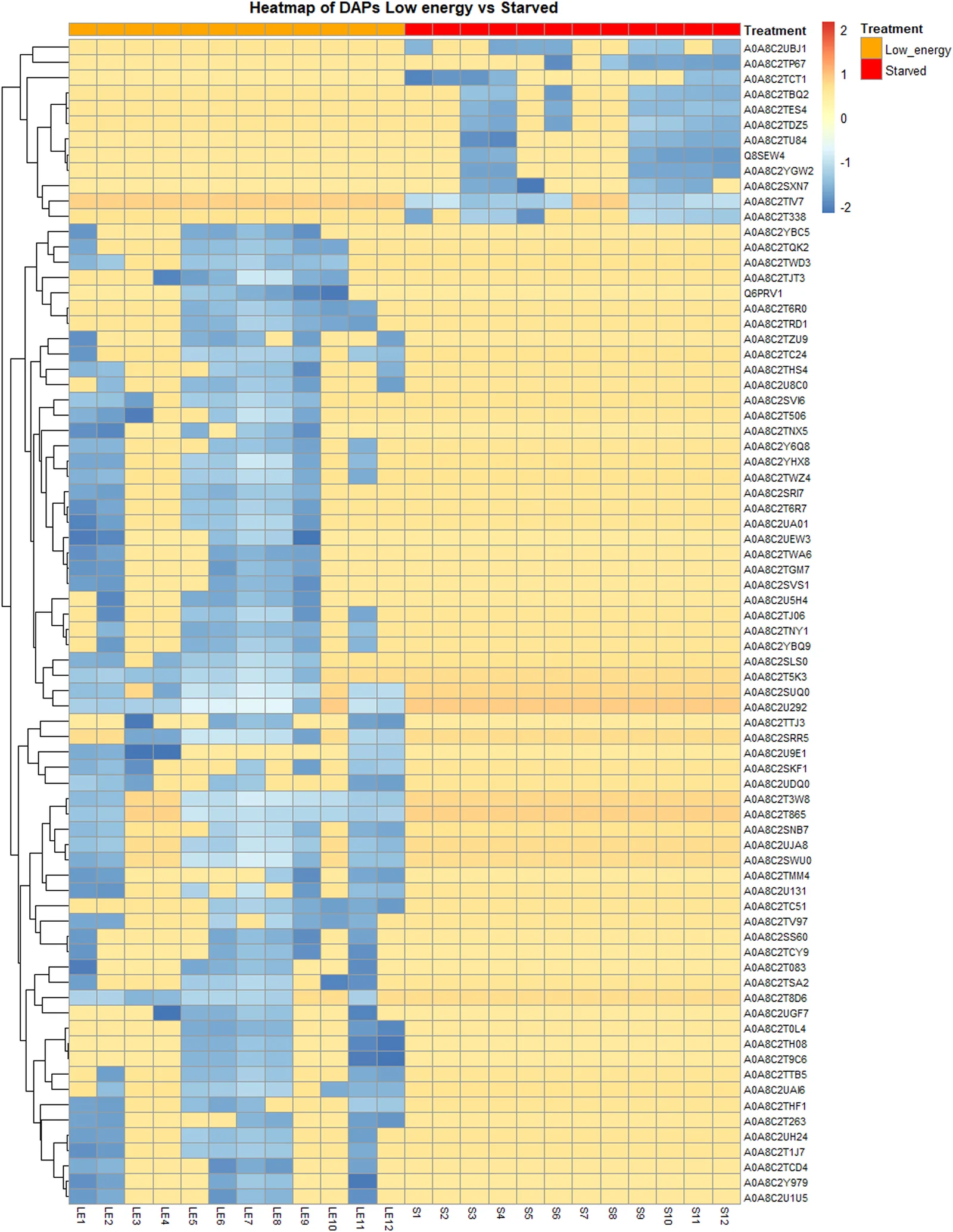
Heatmap of differentially abundant proteins (DAPs) in the low-energy and feed-deprived groups. The expression levels were visualized using a gradient color. Starved: feed-deprived group.

The total number of proteins in the filtered list for the feed-deprived and low-energy groups was 546 (Fig. 9). Of the 76 DAPs between the low-energy and feed-deprived groups, 12 were more abundant in the feed-deprived group, and 64 were more abundant in the low-energy group (Fig. 10, Supplementary Table 2).

**Fig. 11:**
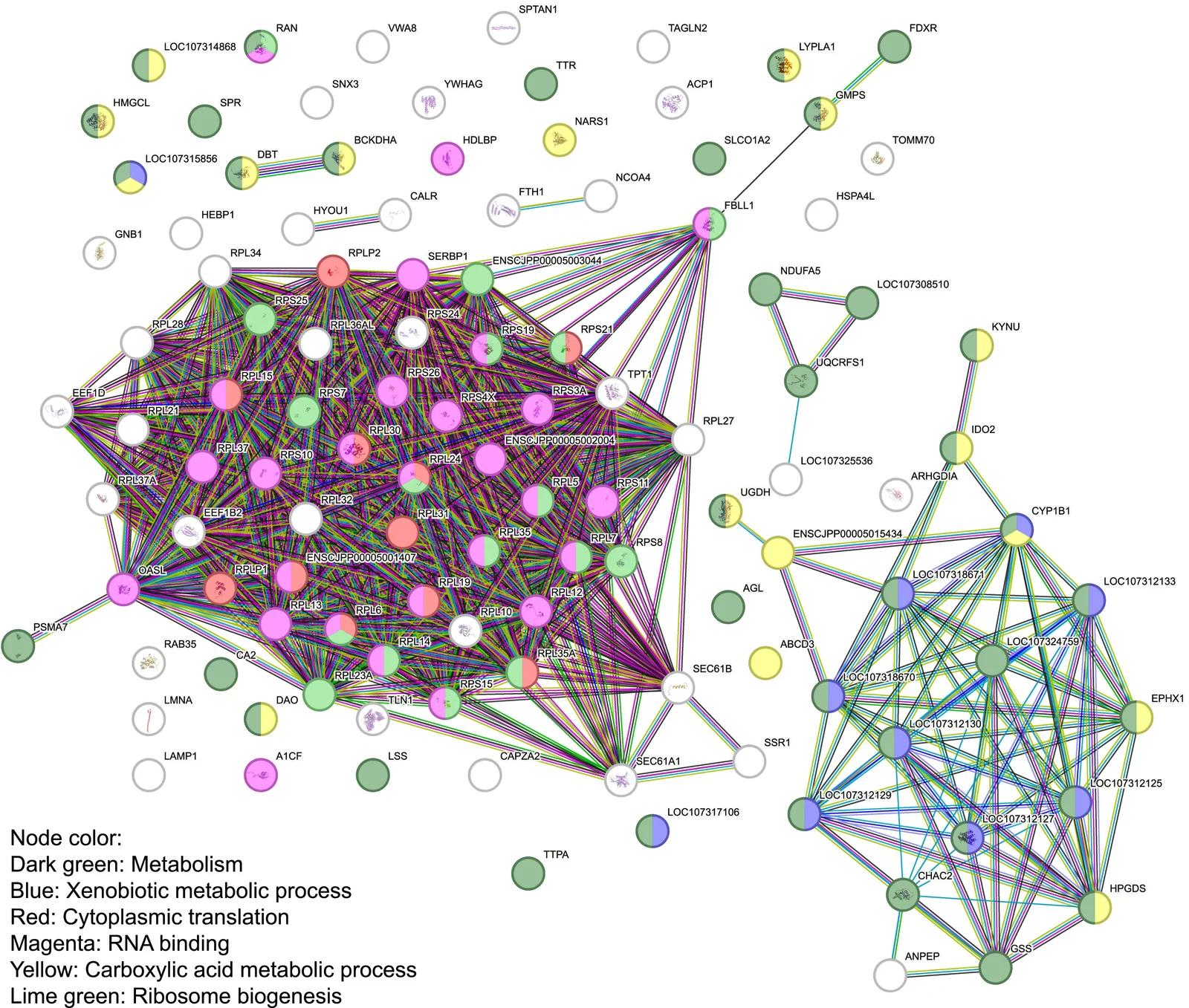
Protein_Protein interaction of differentially abundant proteins with higher abundance in the low-energy group (low-energy vs feed-deprived group comparison) using STRING. Node colors are based on the functions.

The enrichment analysis showed that in the 64 DAPs with higher abundance in the low-energy group, the protein set was enriched for pathways related to cytoplasmic translation (GO:0002181), ribosome biogenesis (GO:0042254), RNA binding (GO:0003723), xenobiotic metabolic process (GO:0006805), carboxylic acid metabolic process (GO:0019752), and metabolism (MAP-1430728) (Fig. 11).

**Fig. 12:**
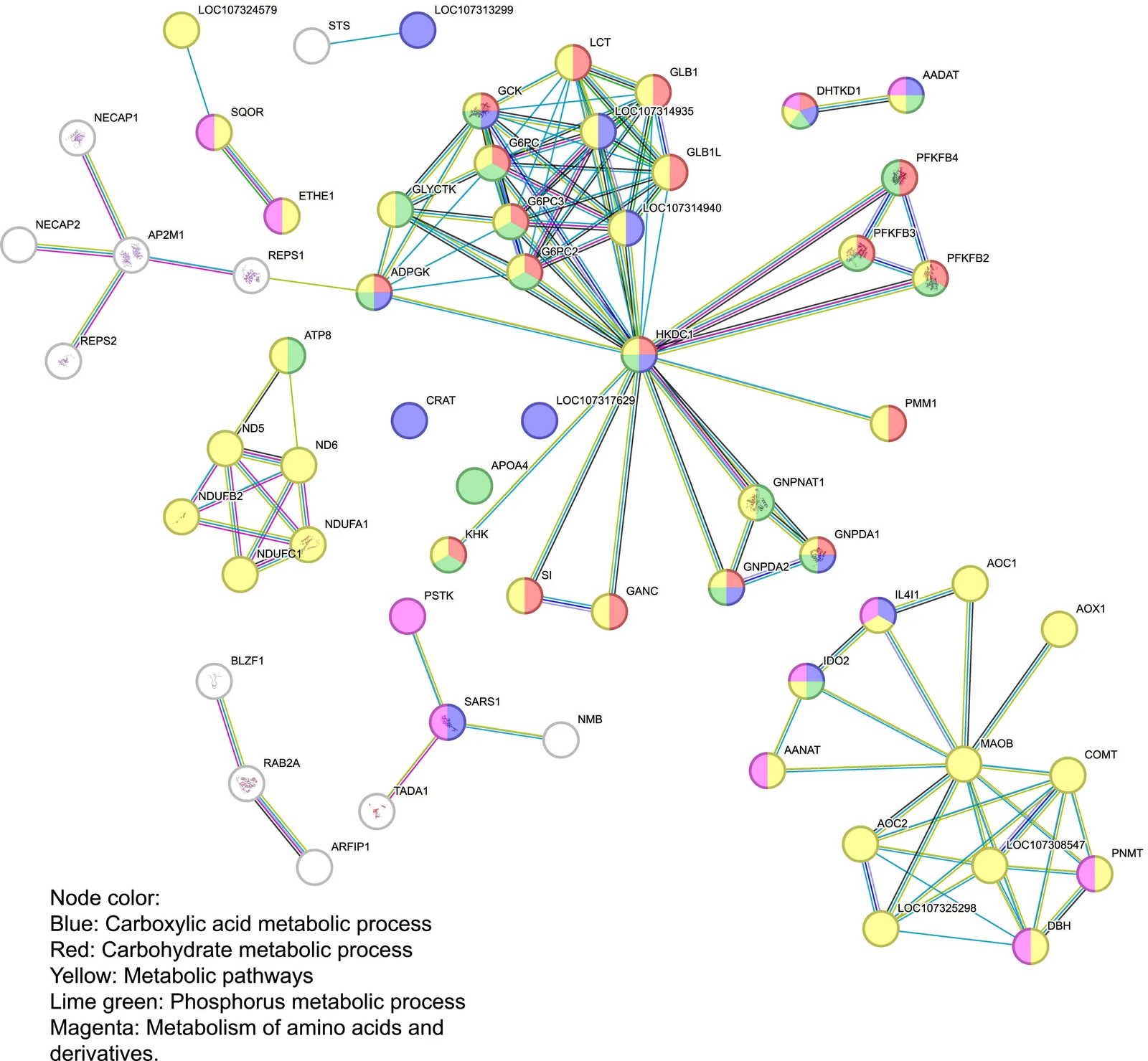
Protein_Protein interaction of differentially abundant proteins with higher abundance in the feed-deprived group (low-energy vs feed-deprived group comparison), using STRING. Node colors are based on function.

In contrast, the 12 DAPs with higher abundance in the feed-deprived group, phosphorus metabolic process (GO:0006793), carbohydrate metabolic process (GO:0005975), carboxylic acid metabolic process (GO:0019752), metabolic pathways (cjo01100), and metabolism of amino acids and derivatives (MAP-71291) pathways, were enriched (Fig. 12).

## 4 Discussion

Monitoring feed intake regulation is commonly performed using the complete deprivation of feed or restricting diets for short periods (Jozsa et al., 2006; Nakashima et al., 2005; NAKASHIMA et al., 2013; Saneyasu et al., 2022; Simon et al., 2017). Serving as a metabolic organ, the liver plays a pivotal role in coordinating nutrient sensing, glucose and lipid metabolism, and endocrine signals related to appetite and energy balance in birds (Denbow, 2015; Zaefarian et al., 2019). In addition, Japanese quail (*Coturnix japonica*) can be a useful avian model for investigating metabolic adaptation mechanisms relevant to poultry species, as they have a fast growth rate, a short reproductive cycle, and are easy to handle.

To understand the DAPs detected, we conducted a functional enrichment analysis to associate the DAPs with their biological functions in each comparison.

### 4.1 Control vs. low-energy

One of the key pathways in the proteins with higher abundance in the low energy group showed increased activity of oxidative phosphorylation, which includes proteins such as NDUFA, NDUFA8, NDUFB8, and NDUFC2 from complex I and SDHB from complex II of the electron transport chain in the inner mitochondrial membrane (Federico et al., 2012; Matsumoto et al., 2012; Midzak & Papadopoulos, 2016). The coordinated enrichment of mitochondrial Complex I and II proteins indicates enhanced oxidative phosphorylation, suggesting that the liver compensates for reduced dietary energy by maximizing mitochondrial ATP production. The concurrent increase in SDHB further supports a metabolic shift toward efficient coupling of the TCA cycle with the electron transport chain (Ricci et al., 2023). This oxidative phosphorylation process is supported by proteins that help manage oxidative stress, such as FDXR and glutathione transferase (Hage-Sleiman et al., 2017; Zhang et al., 2018).

In the enriched metabolism pathway, there are enzymes related to fatty acid beta oxidation and mitochondrial substrate supply proteins, including ACAD11, ACSM5, CPT1A, BCKDHA, HMGCL, and ACOT1-like (Cogburn et al., 2018; He et al., 2011; Li et al., 2023; Mann et al., 2021; Mizuno et al., 2024; Winder & Hardie, 1996). The enrichment of enzymes involved in fatty acid activation, transport, and β-oxidation indicates that lipid catabolism became the primary energy source under reduced dietary energy availability in quails. Similar enrichment of oxidative phosphorylation, and lipid metabolism pathways has also been reported in proteomic analyses of slow-growing chickens with divergent feed efficiency, suggesting that remodeling of mitochondrial energy metabolism is a common molecular feature underlying nutritional adaptation in poultry (Kaewsatuan et al., 2022).

The presence of ribosomal proteins (RPL5, RPL11, RPL12, RPL14, RPL23A, RPL24) and elongation factors (EEF1D, EEF1G) supports these metabolic changes. This suggests that the ability to translate key metabolic and mitochondrial proteins remains active even when energy levels are low (Fakih et al., 2023; Ishii et al., 2006; Xu et al., 2022). Additionally, the increase in RNA-binding proteins (A1CF, HDLBP, HNRNPM, and KARS1) shows that there is selective post-transcriptional regulation, such as the editing, stabilization, and processing of metabolic transcripts (Henkel et al., 1992; Liu et al., 2025; Shi et al., 2020; Wang et al., 2025). These mechanisms facilitate the precise regulation of metabolic protein expression via an energy-sensing system, thereby enabling the liver to respond effectively to reduced energy intake, which is consistent with previous results in goose liver (Zhang et al., 2023a).

From the metabolism-enriched pathway in DAPs with lower abundance in the low-energy group, we found proteins related to glycolysis (HKDC1) and other mitochondrial enzymes, such as Complex IV enzymes in the electron transport chain (COX7A2) and propionate metabolism (MMUT) (Ahmad et al., 2019; Lv et al., 2018; Qu et al., 2021; Xing et al., 2024a).

From other enriched pathways in the control group, basal ribosomal proteins (RPL15, RPL4, EIF4G1), in addition to other proteins related to ER to Golgi anterograde transport (RAB1A, SEC31B, TMED10), were identified, suggesting that cellular protein trafficking and secretion pathways are more active under normal energy conditions and are suppressed under lower energy source availability (Cui et al., 2022; Nakashima et al., 2005; Park et al., 2022; Van Riggelen et al., 2010).

This observation may reflect metabolic adaptation to energy restriction, potentially conserving resources by downregulating the non-essential processes. In the low-energy group, birds maintained selective translation machinery and mitochondrial Complex I/II proteins, supporting enhanced fatty acid oxidation and adaptive redox defense, aligned with liver-specific detoxification processes, which is consistent with the expected adaptations to reduced energy availability (Hu et al., 2016; Reda et al., 2024). Together, these observations indicate a coordinated transition in the liver from basal metabolism to energy conservation under low-energy conditions.

### 4.2 Control vs. feed-deprived group

Among the proteins with higher abundance in the feed-deprived group showing statistically significant differences, there were specific enzymes that helped break down fats (enoyl-CoA delta isomerase 2 (ECI2) and electron transfer flavoprotein subunit (ETFA)). ETFA contributes electrons to the electron transfer respiratory chain, which is derived from the oxidation of fatty acids and amino acids and is used to optimize energy conservation (Garcia Costas et al., 2017). The simultaneous enrichment of proteins involved in mitochondrial electron transfer, ATP transport, and ion homeostasis including ETFA, SLC25A4, and SLC4A1 suggests coordinated optimization of mitochondrial function during nutrient deprivation (Amaz et al., 2025; Bahadoran et al., 2018; Cogburn et al., 2018).

In addition to enhanced oxidative phosphorylation, the feed-deprived liver exhibited coordinated enrichment of proteins involved in fatty acid β-oxidation, indicating a metabolic shift toward lipid utilization as the primary energy source during nutrient deprivation. This response was supported by enzymes involved in fatty acid activation and transport (ACAD11, ACSM5, CPT1A, and DHTKD1), consistent with previous observations in nutritionally challenged poultry (Chen et al., 2021; Zhang et al., 2023b). Alterations in proteins involved in lipid metabolism are consistent with previous avian proteomic studies demonstrating that nutritional interventions modulate enzymes associated with fatty acid metabolism (Zhang et al., 2025). Proteins associated with carboxylic acid metabolism and broader cellular metabolic processes (NPEPPS, CD36, STT3A, HNRNPM, CYP2D6, CYP4A6-like, APOA4, RAB7A, PFKFB2, and ALDH5A1) further suggest increased utilization of alternative metabolic substrates to maintain energy production during fasting. In addition to the enrichment of protein synthesis and handling proteins (EEF1G, KARS1, GARS1, RPL5, RPL11), the identification of PFKFB2 suggests that, despite the suppression of anabolic processes, the liver retained the capacity to regulate glucose homeostasis. PFKFB2 has been implicated in directing carbon substrates toward glucose production under nutrient-limited conditions, supporting metabolic flexibility during acute feed deprivation (Ma et al., 2021).

In addition, proteins involved in xenobiotic metabolism, including CYP2K1-like and GST-like, were strongly upregulated in the feed-deprived group. The coordinated induction of cytochrome P450 and glutathione S-transferase enzymes suggests that acute feed deprivation enhances hepatic detoxification and antioxidant defense mechanisms. During nutritional stress, increased fatty acid oxidation and mitochondrial activity can elevate the production of reactive oxygen species and endogenous metabolites, necessitating greater detoxification capacity to maintain cellular homeostasis (van Vugt-Lussenburg et al., 2022; Zimniak et al., 1999).

In the set of DAPs with higher abundance in the feed-deprived group, proteins showed a shift in metabolism toward using stored fats and organic acids and reusing internal substances for energy, emphasizing that the liver mainly relies on fat breakdown for energy during feed deprivation instead of glucose breakdown.

In contrast to the mitochondrial and lipid oxidative pathway proteins, especially those related to the NADH pathway, observed during feed deprivation , proteins uniquely expressed in the control group were predominantly enriched in pathways related to RNA binding, ribonucleoprotein complex biogenesis, and protein metabolism. The similar enrichment of RNA-binding proteins (e.g., RPS11, RPL15, RPL4, EIF4G1, EEF1B2, RAN) to the previous comparison (low-energy vs. control) suggests that under normal feeding conditions, the liver maintains active translational machinery, ribosomal assembly, and the transport of intracellular proteins, ensuring normal cellular function (Cui et al., 2022; Zhou et al., 2023). Some of these ribosomal proteins, such as RPL4, are involved in the ubiquitination of proteins strongly associated with apoptosis (Li, 2019; Nakashima et al., 2005; Xu et al., 2025; Zhang et al., 2017).

Enrichment of protein metabolic process pathways (FBLL1, CALR, PTGES3, NARS1, CNDP2, ACP1, LYPLA1, ANPEP) in the control group reflected higher basal protein turnover and biosynthetic activity under adequate nutrient availability. Their downregulation during feed deprivation suggests that global protein synthesis is suppressed, which is in line with the energy-conserving tactic in a nutrient-deficient state.

Proteins involved in the carboxylic acid metabolic process and broader metabolism pathways were uniquely enriched in the control group. Many of these proteins (e.g., AGL, PTGES3, CNDP2, KYNU, LSS, CMPK2, DBT, IDO2, and MMUT) are associated with amino acid and lipid metabolism, indicating that the liver actively processes diverse substrates, including amino acids and organic acids, under normal conditions (Cogburn et al., 2020; Fujikawa et al., 2024; Li et al., 2024; Mbongue et al., 2015; Rengaraj et al., 2013; Xu et al., 2008). Among these proteins, steroid receptor chaperone (PTGES3) is involved in appetite regulation in poultry via the secretion of prostaglandins, which may modulate feed intake behavior under normal feeding conditions (Cao et al., 2021; Fu et al., 2013). This pathway was also enriched in the feed-deprived group with a different set of proteins, suggesting that it might be possible that some proteins act only in stress-inducing conditions and others work in normal conditions breaking down nutrients from feed.

Furthermore, the enrichment of xenobiotic metabolic processes (e.g., cytochrome P450 enzymes (CYPs), EPHX1, SLCO1A2) in the control group reflects the routine hepatic processing of dietary compounds and exogenous substrates. In the feed-deprived group, we also observed the induction of specific CYP450 enzymes and GSTs related to the xenobiotic process, which is potentially linked to elevated oxidative stress, which causes the accumulation of endogenous metabolites such as reactive oxygen species (ROS) (Pengsanthia et al., 2025; Wang et al., 2018; Zhou et al., 2013).

Collectively, these findings indicate that feed-deprivation induces the coordinated suppression of ribosomal activity, protein turnover, and normal xenobiotic metabolism pathways in the quail liver. This metabolic reprogramming is accompanied by the observed upregulation of oxidative phosphorylation and fatty acid metabolism, highlighting a clear shift from anabolic and processing functions under fed conditions to energy conservation and mitochondrial optimization during nutrient deprivation.

### 4.3 Low-energy vs. feed-deprived

We observed a clear enrichment of ribosomal proteins (RPS21, RPL6, RPL15, RPL12, RPL7, RPS11, RPL35, RPL23A, RPL14, RPL35A, RPL24) related to ribosome biogenesis, cytoplasmic translation, and RNA binding in the DAPs with higher abundance in the low metabolizable energy group. This upregulation shows that cells actively synthesize proteins despite low-energy availability (Fakih et al., 2023; Ishii et al., 2006). We also observed the enrichment of xenobiotic metabolic processes through cytochrome P450 and detoxification enzymes (HPGDS, CYP1A-like, CYP2H2-like, FMO5-like). The presence of such proteins is expected to result from increased ribosomal activity (Han et al., 2021; Midzak & Papadopoulos, 2016; Zimniak et al., 1999). This regulation might indicate that low-energy conditions trigger a specific detoxification or chemical processing phase that is not present in feed deprivation and completely suppresses protein synthesis to conserve energy in the liver.

Metabolic adaptation in the low-energy group extended beyond mitochondrial respiration and included pathways supporting glycolysis, amino acid catabolism, ketogenesis, fatty acid β-oxidation, and lipid metabolism. Collectively, these pathways indicate that the liver maintained metabolic flexibility by mobilizing multiple energy substrates rather than relying on a single fuel source. This coordinated response was supported by proteins involved in glycolysis (AGL, UGDH) (Clarkin et al., 2011; Liu et al., 2021), oxidative phosphorylation (NDUFC2, NDUFA5, UQCRFS1, FDXR) (Hage-Sleiman et al., 2017), amino acid catabolism (GMPS, IDO2, KYNU) (Fujikawa et al., 2024; Mbongue et al., 2015; Welch & Rudolph, 1982), ketosis (HMGCL, BCKDHA) (Cogburn et al., 2018; Mann et al., 2021), fatty acid β-oxidation (ABCD3), and lipid metabolism (DBT, ACOT1-like) (Cogburn et al., 2020; Li et al., 2023).

Several proteins occupied central positions across multiple metabolic pathways, highlighting the coordinated nature of the hepatic response to feed deprivation. For example, APOA4, DHTKD1, and HKDC1 linked carbohydrate, carboxylic acid, and energy metabolism, whereas CRAT supported fatty acid β-oxidation by facilitating the transport of acetyl groups required for mitochondrial energy production activity (Jiang et al., 2016).

Within the metabolically enriched pathways, we identified MAOB. APOA4 and MAOB link metabolism to feeding behavior by modulating neurotransmitter levels such as serotonin, dopamine, and norepinephrine, which can influence feeding behavior (Bach et al., 1988; Oyarzún et al., 2025).

Systemic glucose homeostasis is maintained by hexokinase domain-containing protein 1 (HKDC1), the absence of which can cause mitochondrial dysfunction, highlighting the importance of protecting mitochondrial function under stress conditions caused by feed deprivation (Lv et al., 2018; Xing et al., 2024b). DHTKD1 (dehydrogenase E1 and transketolase domain-containing protein 1) is involved in amino acid catabolism, which can be used in the TCA cycle (Van Every & Schmidt, 2021).

Proteins enriched in the metabolism pathway also include ND5, which plays a role in oxidative phosphorylation, along with cytochrome P450-related proteins (CYP2D6, CYP4A6-like) and SQOR, which has a role in protecting mitochondria following increased phosphorylation due to increased oxidative stress (Banerjee et al., 2022; Zhang et al., 2023b).

This regulation pattern reflects a metabolic reorientation toward the mobilization of stored lipids (APOA4), carboxylic acids (CRAT, MAOB, DHTKD1) and the recycling of endogenous metabolites for energy production. In addition, increased oxidative phosphorylation involving ND5 was coordinated with the presence of proteins such as SQOR and HKDC1, which help to protect the mitochondria during feed deprivation.

One of the limitations of the study is the small sample size, which may affect statistical power. In addition, these observations highlight the need for future work integrating metabolite profiling, oxidative stress assays, and liver lipid quantification to validate and extend the proteomic findings. Moreover, an important limitation of the present study is that the low-energy diet differed not only in metabolizable energy but also in crude protein concentration (11.6%vs. 18%). Consequently, the observed proteomic changes may reflect combined effects of energy and protein restriction rather than energy restriction alone. The formulation was designed to reduce metabolizable energy primarily through inclusion of sunflower hulls, which inadvertently lowered crude protein content. This approach was intended to mimic practical feed formulation scenarios rather than a strictly controlled isonitrogenous comparison. Future studies should employ isonitrogenous low-energy diets to disentangle the specific contributions of energy restriction from protein restriction.

## 5 Conclusions

Our findings revealed unique state-specific changes in hepatic protein expression in response to feed deprivation and low-energy feeding treatments in quails, emphasizing the central role of the liver in adapting to nutritional stress. Both conditions centrally involve oxidative phosphorylation and lipid utilization, but the low-energy diet maintains selective translation and detoxification capacity to sustain ATP production. In contrast, starvation shifts toward catabolic use of stored substrates, resulting in a broader suppression of ribosomal activity and protein turnover, as well as increased mitochondrial stress management. These results highlight the important role of oxidative phosphorylation and lipid metabolism pathways as primary adaptive mechanisms that function as molecular sensors, offering potential molecular targets for enhancing the feed utilization efficiency and potentially improving nutritional stress tolerance in commercial poultry. These findings remain exploratory and further validation is required to confirm our observations.

## Ethical statement for animal studies

Animal subject: This study was conducted in accordance with the following guidelines for animal welfare and/or reporting: European Union Directive 2010/63/EU. This study was approved by the Ethical Committee for Animal Use at the University of Debrecen, Hungary. (Approval No. Protocol No. 4/2021/DEMAB)

## CRediT authorship contribution statement

**Doha Mohamad Khalifeh:** Writing – review & editing, Writing – original draft, Visualization, Methodology, Investigation, Formal analysis, Data curation, Conceptualization. **Gabriella Gulyás:** Writing – review & editing, Visualization, Validation, Methodology, Investigation. **Renáta Knop:** Writing – review & editing, Validation, Methodology, Investigation, Conceptualization. **Gebrehaweria K. Reda:** Writing – review & editing, Validation, Investigation. **Gergő Kalló:** Writing – review & editing, Validation, Investigation, Data curation. **Csaba Szabó:** Writing – review & editing, Validation, Methodology. **Éva Csösz:** Writing – review & editing, Visualization, Validation, Data curation. **Levente Czeglédi:** Writing – review & editing, Validation, Supervision, Methodology, Conceptualization.

## Declaration of competing interest

The authors declare that they have no known competing financial interests or personal relationships that could have appeared to influence the work reported in this paper.

## Appendix Supplementary materials

Image, application 1

Image, application 2

Image, application 3
