# Supplementary material for: Shotgun proteomic analysis of quail liver under feed deprivation and low-energy diet conditions
Source: Vet Anim Sci. 2026 Aug 11;33:100813. doi: 10.1016/j.vas.2026.100813 (PMC13520742; doi:10.1016/j.vas.2026.100813)
Supplement: Supplementary file 1 [file mmc1.docx]

**Supplementary Figures 1-3**

**Figure 1:** Volcano plot displaying differentially abundant proteins (p-value < 0.05 and log2 Fold Change > 1) between the Low-Energy cohort and the Control group in quail liver.

**Figure 2:** Volcano plot displaying differentially abundant proteins (p-value < 0.05 and log2 Fold Change > 1) between the Starved cohort and the Control group in quail liver.

**Figure 3:** Volcano plot displaying differentially abundant proteins (p-value < 0.05 and log2 Fold Change > 1) comparing the two nutritional challenged groups (Starved vs. Low-Energy) in quail liver.
